# MECHANISMS TO ADDRESS RACIAL/ETHNIC DISPARITIES IN NURSING HOME QUALITY OF LIFE

**DOI:** 10.1093/geroni/igac059.1199

**Published:** 2022-12-20

**Authors:** Tetyana Shippee

**Affiliations:** University of Minnesota, Minneapolis, Minnesota, United States

## Abstract

Evidence documents racial/ethnic disparities in access, quality of care, and quality of life (QoL) among nursing home (NH) residents who are Black, Indigenous and persons of color. Yet, little is known about mechanisms for these disparities. This presentation examines the mechanisms for racial/ethnic disparities in QoL in high-proportion BIPOC facilities while highlighting variability in QoL disparities across these facilities. The presentation uses data from a 5 year mixed-methods project involving 96 resident interviews; 61 staff interviews; and 614 hours of observations in high proportion BIPOC facilities in MN, coupled with resident clinical Minimum Dataset assessments linked with survey data on residents’ QoL. The findings show significant racial/ethnic disparities in QoL with need for system level changes. Given the increasing racial/ethnic diversity of NHs, ensuring equity in QoL for BIPOC residents is an urgent priority for NHs to remain relevant in the future.

